# Learning-Based End-to-End Path Planning for Lunar Rovers with Safety Constraints

**DOI:** 10.3390/s21030796

**Published:** 2021-01-25

**Authors:** Xiaoqiang Yu, Ping Wang, Zexu Zhang

**Affiliations:** 1 School of Astronautics, Harbin Institute of Technology, Harbin 150002, China; 6111820504@hit.edu.cn; 2 China Academy of Space Technology, Beijing 100094, China; dandanping917@163.com

**Keywords:** path planning, learning-based, deep reinforcement learning, lunar rovers

## Abstract

Path planning is an essential technology for lunar rover to achieve safe and efficient autonomous exploration mission, this paper proposes a learning-based end-to-end path planning algorithm for lunar rovers with safety constraints. Firstly, a training environment integrating real lunar surface terrain data was built using the Gazebo simulation environment and a lunar rover simulator was created in it to simulate the real lunar surface environment and the lunar rover system. Then an end-to-end path planning algorithm based on deep reinforcement learning method is designed, including state space, action space, network structure, reward function considering slip behavior, and training method based on proximal policy optimization. In addition, to improve the generalization ability to different lunar surface topography and different scale environments, a variety of training scenarios were set up to train the network model using the idea of curriculum learning. The simulation results show that the proposed planning algorithm can successfully achieve the end-to-end path planning of the lunar rover, and the path generated by the proposed algorithm has a higher safety guarantee compared with the classical path planning algorithm.

## 1. Introduction

As the closest celestial body to the Earth in the universe, the Moon is the main goal of human beings for deep space exploration because of its great location advantage and abundant material resources. From the perspective of the historical development of human deep space exploration, the step-by-step exploration mission of the moon has opened a chapter in human deep space exploration of the universe, and gradually mastered and verified deep space exploration technologies such as orbit, patrolling, and sampling return of extraterrestrial celestial bodies. The scientific exploration of the moon is of far-reaching significance to a series of scientific fields such as deep space exploration and aerospace technology.

With the gradual understanding of the Moon, the main lunar exploration goals of the world’s major aerospace nations in the future will focus on the development and utilization of lunar resources, the establishment of lunar bases, and the way to deep space through the moon. Many countries have formulated ambitious lunar exploration programs for this purpose [1,2,3,4,5]. In the lunar exploration plans of various countries, the lunar rover, as the executive body and an important part of the lunar exploration mission, is the main research object. The future lunar rover will be a multi-functional integrated rover with of all-terrain crossing, resource exploration and utilization, manned exploration and large-scale material transfer capabilities. The movement mode of the rover has gradually changed from remote control and command mode to autonomy and intelligence. The autonomous movement and detection capability of lunar rover will undoubtedly provide a more autonomous and robust detection mode for lunar exploration, and greatly improve the efficiency of lunar exploration.

The path planning technology is one of the most important aspects of the autonomous exploration process as the lunar rover avoids dangerous areas, achieves lunar surface patrol and reaches the target location safely. The rover path planning includes global path planning and local path planning [6]. Global path planning is to plan a path from the starting point to the end point according to the lunar surface topographic map captured by orbiting satellites. The path needs to consider the lunar terrain conditions (such as slope, roughness, etc.), lighting conditions, communication conditions and rover body constraints, and meets the set global optimization index (such as the shortest path, the least energy, etc.) [7,8,9]. The current global planning method has a high computational complexity to find the optimal solution, and due to the limitation of the resolution of the existing lunar map, it can only provide global guidance for the lunar rover. So local path planning is needed to deal with the dynamic unknown environment of the lunar surface. The local path planning is based on the on-board sensor system of the rover to perceive the surrounding lunar environment in real time, reconstruct the terrain and detect obstacles in the area, and then plan a safe obstacle avoidance trajectory that meets the dynamic constraints of the lunar rover [10,11,12]. However, local path planning requires precise reconstruction and sensing of the surrounding environment, and can only achieve very low-speed lunar rover real-time planning due to the processing power limitation of the lunar on-board computer.

In recent years, with the rapid development of artificial intelligence and deep reinforcement learning (DRL) technology, learning-based path planning, obstacle detection, trafficability analysis and other technologies have been widely concerned by researchers [13,14,15]. DRL has the advantages of not requiring environmental maps, strong learning capabilities, and high dynamic adaptability. Its deep network can successfully process high-dimensional information from sensors, and its reinforcement learning mechanism can perform continuous decision-making tasks in complex environments [16,17,18]. The successfully trained network model can directly generate optimal control commands for the lunar rover based on sensor information, omitting the complex environment reconstruction and sensing steps of traditional algorithms, and is very suitable for dynamic planning tasks such as lunar exploration with the unknown environment and limited on-board computing resources. However, most of the current DRL algorithms are developed based on simple environment training such as gym, which are far from being applied in real environments.

In this paper, we propose a learning-based intelligent path planning method for the lunar rover. The main contributions are summarized as follows: (1) Instead of using existing open source training environment or a simple lunar environment designed manually, we built a lunar rover training system by using the Gazebo 3D simulation environment with physical engine, which loads the scaling model of the real terrain data of the moon. And we develop a lunar rover simulator based on jackal unmanned vehicle model, used to simulate the real lunar environment and lunar rover system. (2) We propose a learning-based end-to-end path planning algorithm with safety constraints, in which a safety reward function considering the sliding behavior of the lunar rover is designed, and the sliding rate of the lunar rover is predicted based on the slope angle of the terrain in which the lunar rover is located, and it is used as a reward feedback for the current state to improve the safety assurance of the lunar rover autonomous exploration process. (3) The idea of curriculum learning is used to train the planning network using the lunar surface environment with different terrain features to improve the adaptability of this planner to different terrains on the lunar surface and to achieve a more intelligent autonomous navigation.

The rest of this paper is organized as follows. Section 2 reviews the related work, and Section 3 describes the system environment model. Section 4 introduces the design of DRL method in detail. The simulation results and corresponding comparison are presented in Section 5. The discussion and conclusion is given in Section 6 and Section 7.

## 2. Related Work

At present, the research on the global path planning of lunar rover mostly considers the influence of terrain conditions, illumination conditions, communication conditions and the constraints of rover body, and generates the global path with specified resolution in the specified area according to the optimal cost strategy. Masataku et al. [7] present a comprehensive path-planning method for lunar rover, the constraints of lunar rover’s internal and external elements were considered respectively, and the influence of their different weights on the path was analyzed. According to the different motion characteristics of tracked and wheeled lunar rover, the planned path was evaluated. Genya et al. [19] proposed a path planning and evaluation strategy that explicitly considered the dynamic mobility of lunar rover. Firstly, different paths were generated on the given topographic map with different weight factors, and then all the generated paths were quantitatively evaluated according to the proposed dynamic mobility index, and the most feasible path between them was obtained. Yu et al. [9] proposed a comprehensive global path planning algorithm based on the lunar digital elevation map, and generated a comprehensive smoothness map according to the lunar terrain and illumination conditions. Then the MFA* algorithm is proposed to solve the fast search of large-scale paths on the lunar surface. At present, global path planning can only give global guidance with limited map resolution, and cannot deal with the complex and unknown environment of the real lunar surface.

For the local path planning of lunar rover, Xing et al. [20] proposed a local comprehensive obstacle avoidance planning method based on quantitative evaluation of terrain accessibility and target accessibility, which can achieve local obstacle avoidance and ensure target accessibility. It has been successfully applied to the autonomous navigation of China’s “Yutu” and “Yutu-2” lunar rovers. Masahiro et al. [21] proposed a terrain classification and path planning algorithm that can predict terrain risks, including a machine learning-based terrain classification capable of identifying potential hazards from images, and a risk-aware path planner based on the Rapid Exploration Random Graph (RRG) and A* search algorithms, which can avoid hazards identified by the terrain classifier and explicitly consider the vehicle dynamics constraints. Reiya et al. [10] proposed an RRT* algorithm based on traversability analysis in rough terrain. First, the point cloud data is captured by the LIDAR sensor to form an environmental map, and then the RRT* algorithm is used to sample directly from the LIDAR point cloud data, and the rough terrain accessibility of lunar rover was considered in the process of RRT* tree expansion. Local path planning algorithm needs complex 3D reconstruction and environment perception in advance, which seriously limits the speed of lunar rover. Therefore, it is urgent to develop more intelligent and efficient technology.

The learning-based path planning algorithm has been successfully applied to autonomous navigation of mobile robots in recent years. Gao et al. [22] proposed a new incremental training mode to improve the training speed for the problem of path planning of mobile robots based on deep reinforcement learning (DRL). A path planner (PRM+TD3) that combines the dual-delay depth determination strategy gradient (TD3) with the traditional global path planning algorithm probabilistic road map (PRM) is proposed, which can effectively improve the generalization of the model. Tai et al. [14] proposed a learning-based mapless motion planner, which uses an asynchronous deep reinforcement learning method. The planner can carry out end-to-end training without any manual design features and pre demonstration, and achieve navigation to any target without collision with any obstacles. For learning-based lunar rover planning, Zhang et al. [23] proposed a learning-based global path planning problem for the planetary rover. A new dual branch deep convolution neural network (DB-CNN) is designed and trained. It can directly plan the path from the orbit image of the planet’s surface without performing environment mapping. Masahiro et al. [24] introduced the Machine leaning-based Analytics for Automated Rover Systems (MAARS) proposed by JPL, which aims to bring the latest autonomous driving technology to Mars, the moon and other places. MAARS pays special attention to the following two capabilities: Drive-By Science (DBS) and Risk and Resource-aware AutoNav. It wants to use the most advanced machine learning technology to achieve accurate risk and resource perception to improve the autonomous detection capabilities of the lunar rover. Although the learning-based path planning algorithm can achieve intelligent and efficient autonomous path planning, there are also serious problems such as lack of real training environment, network training difficulties, lack of security assurance, et al. The development of fully autonomous lunar exploration technology is still an ongoing challenge.

## 3. System Model

In this section, we describe the system problem description and the simulation environment for path planning model training.

### 3.1. Problem Description

We formulate the rover path planning problem as a Markov decision process (MDP), which provides a mathematical framework to simulate the stochastic strategies and rewards that agents can achieve in the environment with Markov state, modeled as a tuple (*S*, *A*, *T*, *R*, *γ*). They denote the system’s state set, action set, state transition probability, reward function and discount factor, respectively. The agent’s action selection is modeled as policy π, which is a function mapping from each state *s* ∈ *S* to an action *a* ∈ *A*. The value function *v_π_*(*s*) is defined as the expected sum of discounted rewards starting with the state *s*, and successively taking actions according to π. A policy is called optimal policy π* if it achieves the best *v_π_*(*s*) from any initial state. The goal of deep reinforcement learning is to train a deep neural network which can represent the optimal policy π*, which can select the best action in a given state.

**Figure 1:**
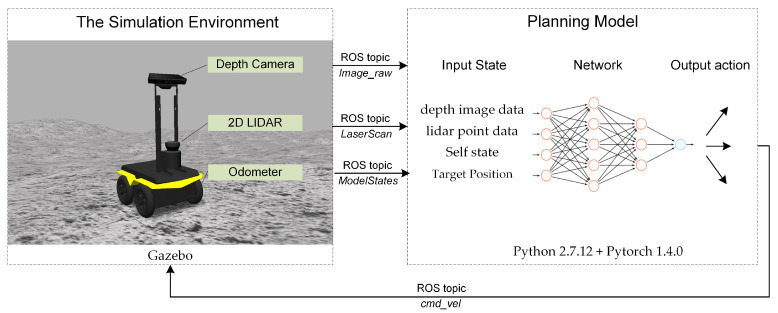
The end-to-end path planner framework.

This paper aims to provide a path planner for autonomous lunar rover exploration, which can perform end-to-end optimal planning based only on sensor information and its own state. The framework of the planner is shown in Figure 1. We are trying to find such an optimal policy as Equation (1):

(1) $$u_{t}{=\pi }_{\vartheta }^{*}\left(s_{t}\right){=\pi }_{\vartheta }^{*}\left(s_{t1}{,s}_{t2}{,s}_{t3}\right)$$

where *s_t_*_1_ is the image data collected by depth camera, *s_t_*_2_ is the range data collected by lidar, and *s_t_*_3_ is the state data of target and lunar rover, policy $\pi_{\vartheta }^{*}$ is a deep neural network parametrized by a parameter vector ϑ.

The purpose of the path planning method in this paper is to guide the lunar rover to find a safe path from the start point to the end point in the complex lunar terrain environment. The implementation details of the planning model are introduced in Section 4.

### 3.2. The Training Environment

At present, most deep reinforcement learning algorithms are trained and verified in a simple simulation environment, especially in the field of autonomous navigation path planning for mobile robots. Most researches are only applied to simple scenarios such as two-dimensional planes or grid maps, and cannot be used for real-world path planning problems. For the autonomous exploration path planning of lunar rover, due to the high risk and value of the lunar exploration mission, the deviation should be avoided as much as possible in the related technology research. Large-scale replication of the lunar environment for actual operations is too expensive, but a feasible alternative is to establish a lunar surface simulation environment, which can reflect the real lunar surface terrain features, so that the training and verification of the lunar rover path planning model is meaningful.

Our goal is to create a lunar surface simulation environment, which can load the real terrain data of the moon, and create the approximate prototype of the lunar rover system in the environment to verify and evaluate the path planning model. Because the learning-based method requires continuous interaction with the environment for trial and error, and hope to realize the functional, end-to-end lunar navigation simulation as soon as possible, it is necessary to establish a simulation framework that provides rich set of off-the-shelf capability.

**Figure 2:**
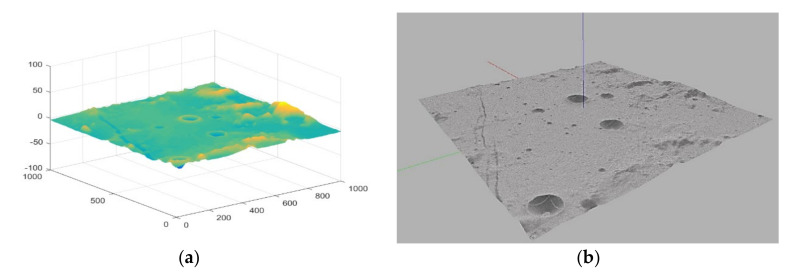
The training environment for lunar rover. (**a**) The scaled DEM elevation map of the simulation scene; (**b**) The simulation scene after loading DEM file and rendering the terrain in Gazebo.

In this paper, the lunar surface simulation environment is established by using Gazebo in the robot operating system (ROS), which is combined with the physical open dynamic engine (ODE) for 3D simulation [25,26]. Similar to the game engine which provides high fidelity visual simulation, Gazebo provides high fidelity physical simulation, which provides a complete set of sensor models and a very user-friendly interaction mode. To generate a more realistic topography of the lunar surface, this paper first downloads the CE2TMap2015 lunar DEM dataset formed by the Chinese Chang’e-2 photography [27], and scales down its elevation data to generate a simulated topography. Select the area with representative features of the terrain near the moon’s equator as the simulation scene. The DEM elevation information of this area is shown in Figure 2a. It can be seen that there are typical lunar features such as large/small craters, mountains, and moon valleys in this area. Load the scaled DEM data of this area as a terrain model into the *.world* file in Gazebo and perform realistic visual renderings of lunar environments, then we can generate a lunar rover simulation training environment as shown in Figure 2b.

### 3.3. The Rover Simulator

In this section, a lunar rover simulator is developed, whose basic motion model is based on the Jackal unmanned vehicle model. It is equipped with 2D lidar and depth camera to detect the lunar environment and simulate driving. The kinematic model of lunar rover is defined as Equation (2).

(2) $$\left\{\begin{array}{c} {\;\dot{x}}_{t}{=V}_{t}{\cos\psi }_{t}\\ {\;\dot{y}}_{t}{=V}_{t}{\sin\psi }_{t}\\ {\;\dot{\psi }}_{t}{=\omega }_{t} \end{array}\right.$$

where (*x_t_*, *y_t_*) is the position of the rover in the two-dimensional Cartesian coordinate system, *V_t_* is the rover’s linear velocity and $\psi_{t}$ is yaw attitude angle, which are updated according to angular velocity ω*_t_*. The other attitude (pitch angle $\theta_{t}$, roll angle $\varphi_{t}$) and the elevation (*z_t_*) of the lunar rover is obtained according to the contact with different terrain and processed by the physical engine of Gazebo. The linear and angular velocity of the lunar rover are updated according to the output of the planning model, which published to the lunar rover model in gazebo through ROS topic, and the frequency of command published is 5 Hz.

**Figure 3:**
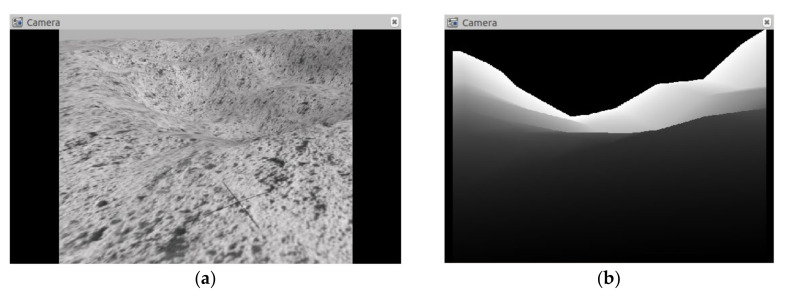
The image results collected and output by the depth camera. (**a**) The topographic map of the moon surface taken by the depth camera; (**b**) The depth image output by the depth camera.

By changing the URDF model file of jackal unmanned vehicle, two kinds of sensors, 2D lidar and depth camera, are added for lunar rover to detect obstacles and potential hazards on the lunar surface. The depth camera uses the Kinect-v1 model to detect potential hazards such as craters and small rocks. Its depth image resolution is 540 × 480, and the detection range is [0.1, 10] m. In order to simplify the model data, the depth image is compressed and stacked the frames into 4 × 80 × 80 dimensional data. The image results of depth camera are shown in Figure 3.

**Figure 4:**
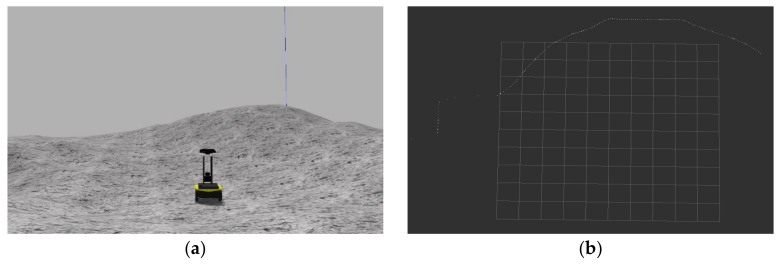
The results collected and output by Lidar. (**a**) The collection scene of the lidar on the lunar surface; (**b**) the distance information output by the lidar.

The lidar uses the lms100 model to detect obstacles such as distant mountains or large rocks. The detection range is set to [−90, 90]°, and the detection range is [0.1, 20] m. The radar output point cloud data is sparsely processed and stacked the frames into 3 × 180 dimensional data, and the update frequency is 50 Hz. The collection scene and output results of lidar are shown in Figure 4.

## 4. Deep Reinforcement Learning

In this section, we introduce the implementation details of deep reinforcement learning, and formulate state space, action space, network structure, reward function, and training method respectively.

### 4.1. State Space

In this paper, the path planning problem of lunar rover is described as Markov decision process (MDP). The information observed by lunar rover sensors and the position and state information of the target relative to itself are taken as its own state. At time t, the state **s***_t_* is composed of depth image data *s_t_*_1_ (4 × 80 × 80 dimensions), lidar point cloud data *s_t_*_2_ (3 × 180 dimensions) and state information *s_t_*_3_ (1 × 4 dimensions) of target and itself respectively. Where state *s_t_*_3_ includes the angle and distance between the rover and target point, the linear and angular velocity at the previous time step. In order to improve the adaptability of the planning model to different scale mobile planning, the state information is normalized, and the missing data in the depth image and lidar data is replaced by the maximum detection range.

### 4.2. Action Space

We take the linear velocity *V_t_* and angular velocity ω*_t_* commands of the lunar rover as the action **a***_t_* to control the rover’s motion. In order to facilitate the network decision, we discretize the action **a***_t_* into 10 values, and the corresponding situations are shown in Table 1.

### 4.3. Network Architecture

**Figure 5:**
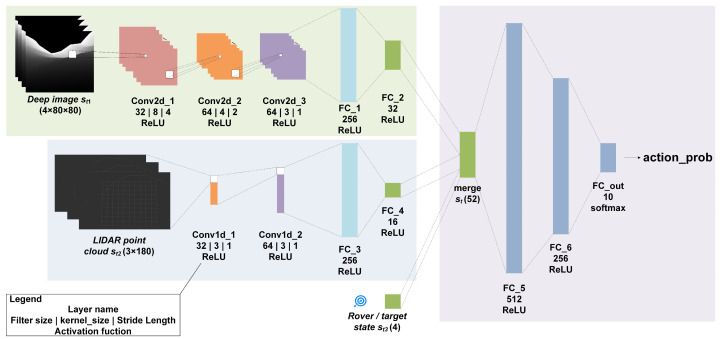
The deep neural network architecture.

We use CNN to extract environmental features from sensor information, and then use deep neural networks to perform nonlinear approximation of RL value and policy functions to realize the path planning of the lunar rover. The network structure is shown in Figure 5. The network input is the state defined in Section 4.1, and the output is the selection probability of action value defined in Section 4.2.

As mentioned above, the input state consists of depth image information *s_t_*_1_, 2D LIDAR point cloud data *s_t_*_2_ and lunar rover/target status *s_t_*_3_. The network mainly includes three blocks. First, a three-layer two-dimensional CNN is used to preprocess the depth image, and the terrain features are extracted through three-layer convolution and ReLU activation and a two-layer fully connected network. Then use two-layer one-dimensional convolutional network and full connection to extract the obstacle feature from the lidar point cloud. Finally, the pre-processed states are merged, a two-layer hidden layer fully connected network is used to realize the mapping of states to actions, and *softmax* is used to realize the output distribution of action value.

### 4.4. Reward Function

The previous section described the input, output and network structure of the DRL-based planning model. Next, we introduce how to design the reward function of the planning model. The reward function is a key element in DRL, the only feedback the model obtains from the environment, and the learning orientation of the model. It determines the ability of the model to learn and the efficiency of the model. For the path planning problem of autonomous exploration of the lunar rover, the goal of learning is to make the lunar rover safely move from the starting point to the end point. In the process of autonomous lunar rover detection, the safety of the lunar rover is the most important factor for the success of its exploration mission, which must be considered in the process of movement planning. The soft and easy-slip soil conditions on the lunar surface are likely to cause the lunar rover to slip. The lunar rover should avoid hazards such as wheel slipping, collision with obstacles, or rollover on inclined terrain to ensure the safe and stable operation of the lunar rover. Considering the above situation, the reward function is designed as shown in Equation (3):

(3) $$r^{t}{=r}_{\mathrm{dis}}^{t}{+\alpha }_{s}{\times r}_{\mathrm{safe}}^{t}$$

where $r_{\mathrm{dis}}^{t}$ and $r_{\mathrm{safe}}^{t}$ respectively correspond to the distance and safety instant rewards of the lunar rover at time t. $\alpha_{s}$ is the safety factor, which can be adjusted according to the terrain.$r_{\mathrm{dis}}^{t}$ is defined as Equation (4):

(4) $$r_{\mathrm{dis}}^{t}=\left\{\begin{array}{c} \begin{array}{cc} 10, & {\mathrm{if}(d}_{p}^{t}{<d}_{\mathrm{goal}}) \end{array}\\ \begin{array}{c} \begin{array}{cc} -10, & {\mathrm{if}(d}_{o}^{t}{<d}_{\min}) \end{array}\\ \begin{array}{cc} d_{p}^{t-1}{-d}_{p}^{t}, & \mathrm{otherwise} \end{array} \end{array} \end{array}\right.$$

where $d_{p}^{t}$ and $d_{p}^{t-1}$ represent the real-time distance of the lunar rover from the target point at time *t* and *t* − 1, $d_{o}^{t}$ represents the distance from the nearest obstacle at time *t*, which is the minimum value of the lidar point cloud data, the threshold distance $d_{\mathrm{goal}}$ and $d_{\min}$ determines whether it reaches the target point or encounters an obstacle. By reward $r_{\mathrm{dis}}^{t}$, the rover can be guided to reach the target point gradually. In addition, this article defines $r_{\mathrm{safe}}^{t}$ as Equation (5) to prevent the lunar rover from slipping, rollover, etc.

(5) $$r_{\mathrm{safe}}^{t}=\left\{\begin{array}{c} \begin{array}{cc} -10, & {\mathrm{if}(\theta }_{t}{>\theta }_{\max}) \end{array}\mathrm{or}(\varphi_{t}>\varphi_{\max})\\ \begin{array}{cc} {-(\mathrm{slip}}_{t}*\beta_{t}), & \mathrm{otherwise} \end{array} \end{array}\right.$$

where $\theta_{t}$ and $\varphi_{t}$ are the real-time pitch and roll attitude angles of the lunar rover, $\theta_{\max}$ and $\varphi_{\max}$ are the maximum safe attitude angle of the lunar rover which is limited to ensure the safety of the autonomous exploration of the lunar rover. ${\mathrm{slip}}_{t}$ is the lunar rover slip rate, and $\beta_{t}$ is the lunar rover slip angle, which can be defined as a function of the attitude angle of the lunar rover according to the literature [7], as shown in Equation (6):

(6) $$\begin{array}{l} {\mathrm{slip}}_{t}{=\mathrm{Ae}}^{{B\theta }_{t}}\\ \beta_{t}{=\mathrm{Ce}}^{D\varphi_{t}} \end{array}$$

where *A*, *B*, *C*, *D* is a constant determined by the motion behavior of the lunar rover on a given terrain. According to the experimental data in the literature [7], for the wheeled lunar rover, the constants are as follows: *A* = 0.07, *B* = 0.10, *C* = 1.32, *D* = 0.16.

In view of the definition of the reward function, the reward $r_{\mathrm{dis}}^{t}$ can guide the lunar rover to gradually reach the target point, and the reward $r_{\mathrm{safe}}^{t}$ can ensure the safe and stable movement of the lunar rover, and realize the safe autonomous path planning of the lunar rover.

### 4.5. Training Algorithm

This paper uses the proximal policy optimization (PPO) algorithm to train the deep neural network planning model, which is a new type of policy gradient (PG) algorithm [28]. The PG algorithm selects an action through observation information and directly conducts back propagation, and uses rewards to directly enhance and weaken the possibility of selection action. The PG algorithm is very sensitive to the step size, but it is difficult to choose an appropriate step size. If the difference between the old and new strategies is too large during the training process, it is not conducive to learning. PPO proposes a new objective function that can be updated in multiple training steps in small batches, which improves the stability and convergence of the algorithm. The main improvements include two points.

Firstly, in the parameter update process, the generalized advantage estimation is defined to reduce the estimated variance instead of the action-value function, as shown in Equation (7):

(7) $${\;\hat{A}\;}^{\pi }{(s,a)=\hat{Q}}^{\pi }{(s,a)-V}^{\pi }\left(s\right)$$

where ${\hat{Q}}^{\pi }\left(s,a\right)$ is the estimated action-value function, $V^{\pi }\left(s\right)$ is the approximation of the state-value function.

Secondly, PPO algorithm constructs an unconstrained surrogate objective function to avoid large-scale policy updates, and uses *clip* function to limit the updating parameters in a certain trust domain. The objective function is defined as Equation (8):

(8) $$J_{\mathrm{ppo}}(\vartheta {)=E}_{s,a}\left[\min\left(\rho_{\vartheta }A^{\pi_{\vartheta }{}_{\mathrm{old}}},\mathrm{clip}\left(\rho_{\vartheta },1-\varepsilon ,1+\varepsilon \right)A^{\pi_{\vartheta }{}_{\mathrm{old}}}\right)\right]$$

where $\rho_{\vartheta }=\pi_{\vartheta }(a\left|s\right.)/\pi_{\vartheta_{\mathrm{old}}}(a\left|s\right.)$, ε controls the size of the trust region for update. Through these two improvements, the PPO algorithm has realized a stable update process.

The network training process is shown in Algorithm 1. First, three networks are initialized: two policy networks (the old actor $\pi_{\vartheta_{\mathrm{old}}}$ and the new actor $\pi_{\vartheta }$) and one critic network $V_{Ϛ}$ (Line 1). In one episode, the agent first uses the new actor network $\pi_{\vartheta }$ to interact with the environment to obtain a batch of data, and use the critic network to obtain the estimated value function. Then, according to the value function estimated by the critic network and the reward of each moment stored in the batch data, the value function of each moment in the collected batch data is calculated according to a certain discount rate (Line 3–4). We set the size of the memory pool to 1000 samples. If the number of samples stored in the pool exceeds this number, the network is trained with randomly selected samples in the pool. Then use the TD error of the batch data to optimize the parameters of the new actor network $\pi_{\vartheta }$ for $K_{\vartheta }$ times (lines 6–9), The optimization tool used is Adam optimizer [29]. Finally, use the same method to optimize the critic network $V_{Ϛ}$ for $K_{Ϛ}$ times (lines 10–13).**Algorithm 1**: PPO-based training algorithm for path planning1Initialize policy network $\pi_{\vartheta }$, $\pi_{\vartheta_{\mathrm{old}}}$ and value function $V_{Ϛ}$, let $\pi_{\vartheta }$=$\pi_{\vartheta_{\mathrm{old}}}$
2**for** episode = 1,2,… N, do3 Run policy $\pi_{\vartheta }$ for T timesteps, collecting experience {**s***_t_*, **a***_t_*, *r_t_*}4 Estimate advantages using ${\;\hat{A}}_{t}{=\delta }_{t}{+(\gamma \lambda )\delta }_{t+1}{+L+L+(\gamma \lambda )}_{T-t+1}\delta_{T-1}$, where $\delta_{t}=r_{t}+\gamma V\left(s_{t+1}\right)-V\left(s_{t}\right)$
5 **if** Memory_size > 1000, do6  **for**
*j* = 1,2,…$K_{\vartheta }$, do7   
$J_{\mathrm{ppo}}(\vartheta )=-E_{s,a}\left[\min\left(\rho_{\vartheta }^{t}{\;\hat{A}}_{t},\mathrm{clip}\left(\rho_{\vartheta }^{t},1-\varepsilon ,1+\varepsilon \right){\;\hat{A}}_{t}\right)\right]$
8   Optimize surrogate $J_{\mathrm{ppo}}(\vartheta )$ wrt ϑ, with $K_{\vartheta }$ epochs, minibatch size B_s_ and the learning rate $l_{r\vartheta }$
9  **end**10  **for**
*j* = 1,2,…,$K_{Ϛ}$, do11   
$L_{V}(Ϛ)=-\sum_{t=1}^{T}(\sum_{t\prime >t}\gamma^{t\prime -t}r_{t\prime }-V_{\vartheta \prime }(s_{t}){)}^{2}$
12  Optimize surrogate $L_{V}(Ϛ)$ wrt Ϛ, with $K_{Ϛ}$ epochs, minibatch size B_s_ and the learning rate $l_{rϚ}$
13  **end**14  Clear the memory pool15 **end**16end


**Figure 6:**
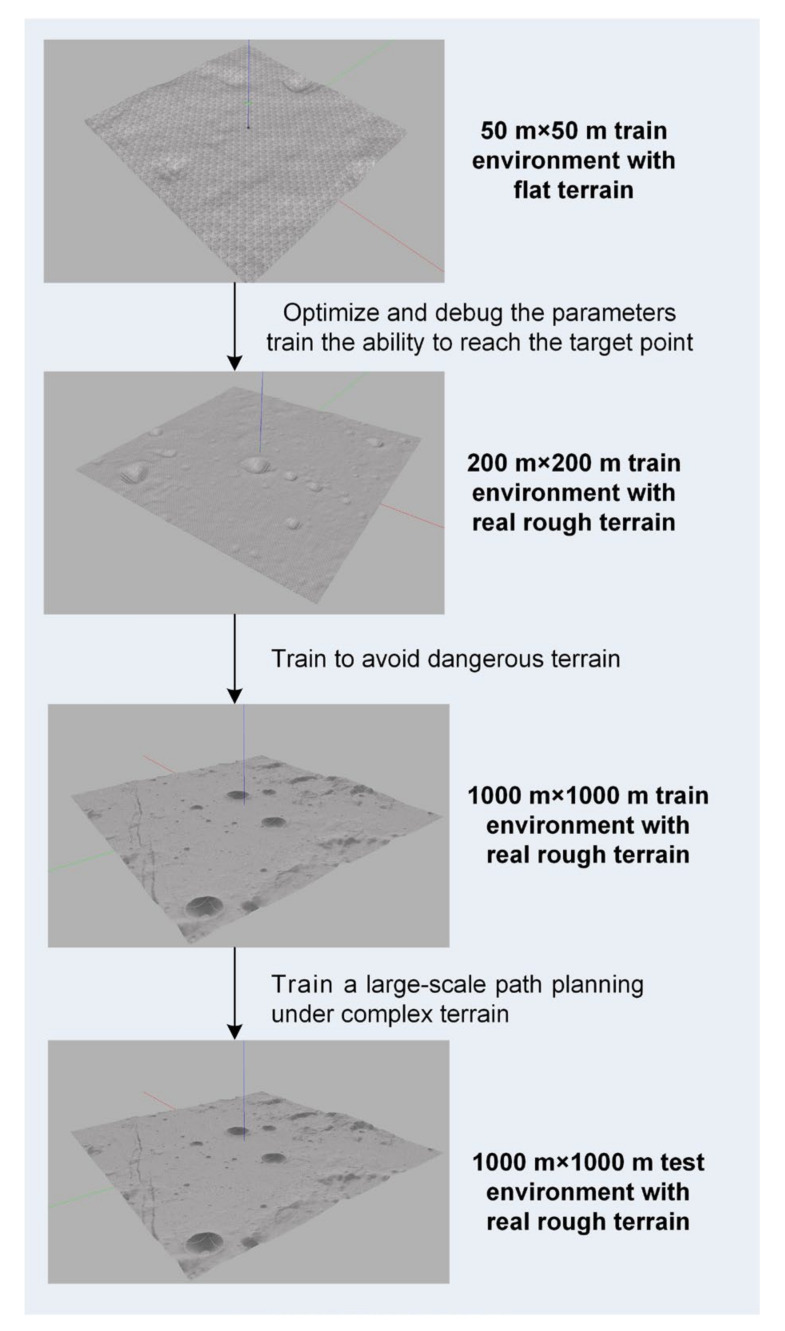
Different training scenarios in the process of curriculum learning.

In addition, in order to improve the adaptability of the planner to different terrains and different scales of movement planning on the lunar surface, this article adopts the idea of curriculum learning and uses different terrain features and different sizes of lunar environment training planning networks [30]. The curriculum learning process is shown in Figure 6. First, train on a small area with a very flat terrain, optimize and debug the algorithm parameters and train the ability to reach the target point in a simple scenario. Then gradually increase the scope of the planning scene and the complexity of the terrain in the scene, and gradually increase the safety reward factor, so that it will gradually learn a large-scale path planning under complex terrain and realize more intelligent autonomous navigation.

## 5. Simulation Results and Analysis

In this section, we perform the simulation validation of the proposed method. First, the simulation settings and parameters are described, and then the training results under different scenarios are presented. Finally, we present a comparative analysis of our method with two other typical methods.

### 5.1. Simulation Setup

This paper sets up three training scenarios from simple to difficult. The scene 1 is a 50 m × 50 m flat terrain environment. The starting point of the lunar rover is set at a random position within 5 m of the origin, and the end point is set at a random position 20 m away from the starting point. The scene 2 is a 200 m × 200 m environment with a complex terrain, while scene 3 is a 1000 m × 1000 m environment with real terrain, and the planning distance is set to 50 m and 200 m respectively. Different scenarios and planning distances can make the distribution of samples wider and improve the generalization ability of the planning network. The parameters of the three scenarios and PPO network training parameters are shown in Table 2. 

In this paper, the deep neural network is implemented in Python 2.7.12 with Pytorch 1.4.0 [31] + CUDA 10.1. The simulation environment is based on ubuntu16.04 + ROS kinetic + gazebo 2.7.16. The hardware environment is an Intel(R) core(TM) i9-9820X CPU + 16.0 Gb RAM + an NVIDIA GeForce GTX 1080ti GPU.

It is worth noting that the default value of *real_time_update_rate* in Gazebo is 1000, and the default value of *max_ step_size* is 0.001. Multiplying the two to obtain the ratio of simulation time to real time *real_time_factor* defaults to 1. In order to speed up the simulation speed, modify the value of *max_step_size* = 0.005 in *.world* file of the simulation environment, so that can make *real_time_factor* = 5 can be used to speed up the simulation speed by 5 times.

### 5.2. Training Results in Different Scenarios

**Figure 7:**
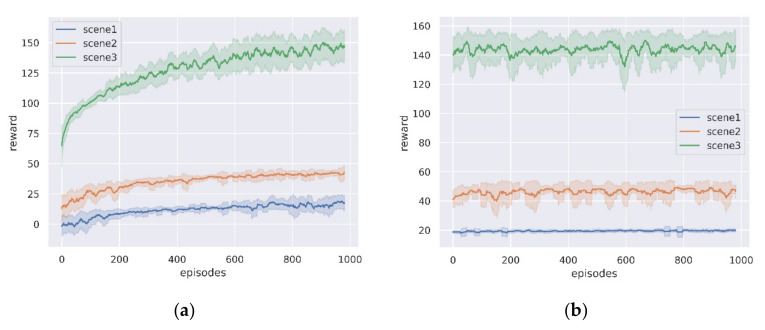
The average reward curve in the three simulation scenarios. (**a**) Training process; (**b**) Evaluation process.

In this subsection, we analyze the results for the three training scenarios. Figure 7a shows the variation of the cumulative rewards during training for the three scenarios, and we record the average of the total rewards per 50 episodes. As can be seen from the figure, the rewards gradually increase and converge as the training episodes increase, indicating that the lunar rover gradually learns to take high reward actions. Figure 7b shows the evaluation of the trained network in three scenarios, also tested for 1000 episodes. It can be seen that stable performance can be achieved in all three scenarios, indicating that the planning network model can solve the path planning problems in different scales and terrain environments, and the performance of the three scenarios tested is shown in Table 3.

**Figure 8:**
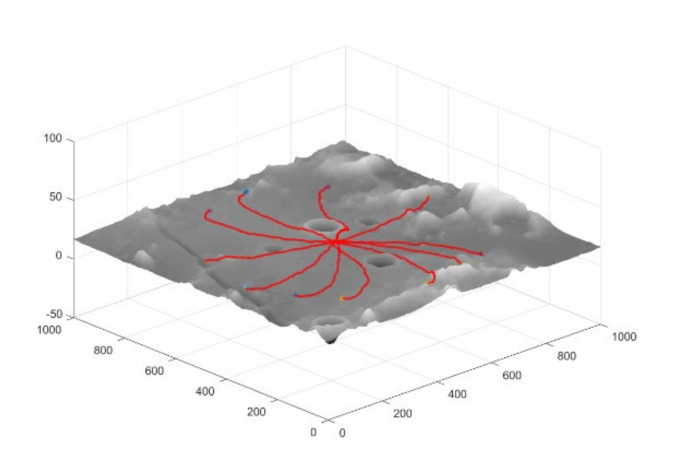
Partially successful path to the target point in scenario 3.

As can be seen from the data in Table 3, the lunar rover can achieve a high rate of successful arrival at the target point. However, in Scenario 3, due to the existence of large craters or mountains in the environment, and the limited detection distance of onboard sensors of the lunar rover cannot guide the lunar rover to avoid hazards, leading to a decrease in the success rate of planning. Similarly, path length and slip rate also increase with terrain complexity. In addition, in order to verify the adaptability of the planning network in a larger-scale planning problem, we set the end-point distance to 400 m in scenario 3, and part of the path is shown in Figure 8. It can be seen that the planning model can successfully achieve large-scale path planning in the real lunar terrain environment.

### 5.3. Comparison Results with Other Algorithms

In this section, we compare our trained planning model in a large-scale path planning scenario with two other classical models, the heuristic search-based A* algorithm and the value-based deep reinforcement learning DQN algorithm [32,33]. Among them, DQN uses the same network structure and training process as in this paper, and the training parameters are shown in Table 4. And the A* algorithm uses the terrain processing method proposed in the literature [9] to pre-convert the elevation map into a traversability binary map and search for the optimal path in the feasible region.

**Figure 9:**
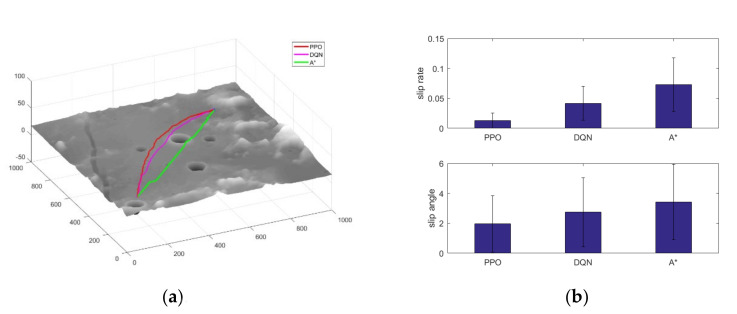
Comparison of the three algorithms. (**a**) Path comparison; (**b**) Slip control comparison.

Figure 9a shows the paths planned by the three algorithms, where the path generated by the A* algorithm leads directly to the target point, avoiding obstacle areas on the way that exceed the capability limit of the lunar rover. While the trajectories generated by the PPO and DQN algorithms can be relatively far away from the danger areas. Figure 9b shows the changes of slip rate and slip angle of the paths generated by the three algorithms, and it can be seen that the paths planned by the algorithm proposed in this paper have obvious advantages in terms of slip rate and slip angle control, which can improve the safety of the lunar rover autonomous exploration process.

**Figure 10:**
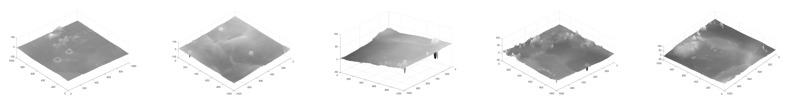
The scenarios for planning algorithm adaptability test.

In order to test the adaptation of the planning algorithm in different scenarios, this section creates five other scenarios for the algorithm generalization test. The test scenarios are shown in Figure 10. In each scene, select 10 sets of starting points and ending points with a distance of more than 500 m. The performance of the paths generated by the three algorithms is statistically presented in Table 5. Although the path length of the algorithm proposed in this paper has increased, it has advantages in the control of the slip rate of the lunar rover, as well as the maximum attitude during the driving process. In addition, the learning-based planning algorithm proposed in this paper can realize end-to-end planning relying on sensor data without global map information, which can realize adaptive path planning with a higher safety guarantee for the lunar rover.

## 6. Discussion

The algorithm proposed in this paper enables end-to-end path planning for lunar rovers with safety constraints. Compared with traditional planning algorithms such as A*, it does not require global map information and the search and optimization process of the optimal path, and it can learn the optimal planning strategy by interacting with the environment. Meanwhile, compared with the current local planning algorithms, its end-to-end planning framework can realize giving lunar rover movement commands based on sensor information without precise environment reconstruction and obstacle detection, which can save a lot of computational resources and time. In addition, the algorithm proposed in this paper can effectively reduce the slip phenomenon during the movement of the lunar rover, and can improve the safety of the autonomous detection of the lunar rover.

However, there is still a lot of work to continue improving this paper. First, the simulation training environment constructed in this article only considers the real lunar terrain, and the real space environment has very harsh and challenging environments such as illumination, electromagnetics, and temperature, which have a great impact on the lunar rover sensor and motion system. How to consider these constraints to construct as complete a space simulation environment as possible is an ongoing challenge. In addition, it can be seen from the results in Section 5.2 that due to the limitation of the detection range of the lunar rover’s on-board sensor, it is sometimes impossible to effectively avoid obstacles such as large craters. In this case, the idea of hierarchical planning can be adopted, combined with the global path planning algorithm based on map information, to plan the path points in advance, and use the planning algorithm proposed in this article to realize real-time path planning between global path points and realize large-scale lunar exploration planning, which will be the focus of subsequent attention in this paper.

## 7. Conclusions

Aiming at the problem of autonomous exploration path planning for lunar rover, this paper proposes a learning-based end-to-end path planning algorithm with safety constraints. Firstly, a lunar rover training environment was built using Gazebo simulation environment, which loads a scaled-down model of real lunar terrain data, and a lunar rover simulator was developed based on the Jackal unmanned vehicle. Then the end-to-end path planning algorithm is designed based on PPO deep reinforcement learning algorithm, and the design methods such as state space, action space, network structure, reward function, and training method are proposed respectively. Among them, the safety reward function is designed considering the sliding behavior of the lunar rover, and the sliding rate of the lunar rover is predicted based on the slope angle of the terrain it is on and the current state of the lunar rover, and it is used as the reward feedback of the current state. In addition, the idea of curriculum learning is used to train the network using lunar surface environments with different terrain features to improve the adaptability of this planning algorithm to different terrains on the lunar surface. Finally, we compared with two classic algorithms, DQN and A*, and the results show that the algorithm proposed in this paper can realize end-to-end autonomous detection path planning, and the generated path has a higher safety guarantee.

## Figures and Tables

**Table 1:** Correspondence table of lunar rover discrete action value and speed command.

Action Value	Linear Velocity (m/s)	Angular Velocity (rad/s)
0	1	1
1	1	0.5
2	1	0
3	1	−0.5
4	1	−1
5	0.5	1
6	0.5	0.5
7	0.5	0
8	0.5	−0.5
9	0.5	−1

**Table 2:** Training parameters in Algorithm 1.

Parameters	Values	Descriptions
T	100 (scene 1) 300 (scene 2) 3000 (scene 3)	The maximum timesteps in one episode
N	1000	The total episode
$\alpha_{s}$	0.5 (scene 1) 1 (scene 2) 1 (scene 3)	The safe factor
γ	0.99	The reward discount
λ	0.95	Generalized advantage estimation parameter
ε	0.1	Clip range
$K_{\vartheta }$	10	The actor network training times
$K_{Ϛ}$	10	The critic network training times
B_s_	64	Minibatch size
$l_{r\vartheta }$	0.00002	The actor network learning rate
$l_{rϚ}$	0.001	The critic network learning rate

**Table 3:** Evaluation performance of three simulation scenarios.

Performance Item	Scene1	Scene2	Scene3
The success rate (%)	99.5	95.1	88.6
Average path length (m)	21.35	53.48	217.32
Average slip rate	0.0122	0.0165	0.0266
Average slip angle (°)	1.1178	1.5815	1.9710

**Table 4:** Training parameters of the DQN.

Parameters	Values	Descriptions
T	100 (scene 1) 300 (scene 2) 3000 (scene 3)	The maximum timesteps in one episode
$\alpha_{s}$	0.5 (scene 1) 1 (scene 2) 1 (scene 3)	The safe factor
γ	0.99	The reward discount
Bs	64	Minibatch size
$l_{q}$	0.0005	The learning rate
Ms	10000	The replay memory size

**Table 5:** Comparison of the path performance of the three algorithms.

Performance Item	PPO	DQN	A*
Average path length (m)	845.78	851.66	691.54
Average slip rate	0.0276	0.0528	0.0844
Average slip angle (°)	2.0648	2.9362	3.6952
Maximum pitch angle (°)	13.3583	17.8628	18.575
Maximum roll angle (°)	8.4252	8.5486	13.5572

## Data Availability

No new data were created or analyzed in this study. Data sharing is not applicable to this article.
